# Impact of metabolic syndrome and metabolic dysfunction-associated fatty liver disease on cardiovascular risk by the presence or absence of type 2 diabetes and according to sex

**DOI:** 10.1186/s12933-022-01518-4

**Published:** 2022-06-02

**Authors:** Yasuhiro Matsubayashi, Kazuya Fujihara, Mayuko Yamada-Harada, Yurie Mitsuma, Takaaki Sato, Yuta Yaguchi, Taeko Osawa, Masahiko Yamamoto, Masaru Kitazawa, Takaho Yamada, Satoru Kodama, Hirohito Sone

**Affiliations:** Department of Hematology, Endocrinology and Metabolism, Faculty of Medicine, Niigata University, 1-754 Asahimachi-street, Niigata-city, Niigata 951-8510 Japan

**Keywords:** Metabolic syndrome, Metabolic dysfunction associated fatty liver disease, Fatty liver, Cardiovascular disease, Type 2 diabetes

## Abstract

**Background:**

To determine the impact of metabolic syndrome (MetS) and/or metabolic dysfunction-associated fatty liver disease (MAFLD), which are pathophysiologically similar and include insulin resistance, on the development of new-onset cardiovascular disease with and without type 2 diabetes and according to sex.

**Methods:**

This study included 570,426 individuals without a history of cardiovascular disease who were enrolled in a nationwide claims database from 2008 to 2016 and were classified by the presence or absence of MetS and/or MAFLD stratified by the presence or absence of type 2 diabetes and sex. The fatty liver index was used to determine the presence or absence of fatty liver that required a diagnosis of MAFLD. Risks of developing coronary artery disease (CAD) and cerebrovascular disease (CVD) in each category were analyzed using a multivariate Cox proportional hazard model.

**Results:**

During a median follow-up of 5.2 years, 2252 CAD and 3128 CVD events occurred. Without type 2 diabetes the hazard ratio (HR) (95% CI) for CAD/CVD compared with neither MAFLD nor MetS was 1.32 (1.17–1.50)/1.41(1.28–1.57) for MAFLD only (without MetS), 1.78 (1.22–2.58)/1.66 (1.34–2.06) for MetS only (without MAFLD), and 2.10 (1.84–2.39)/1.73 (1.54–1.95) for MAFLD + MetS. For those with type 2 diabetes, the HR for CAD for MAFLD only (compared with neither MAFLD nor MetS) was 1.29 (1.06–1.58), for MetS only 1.34 (0.84–2.13), and for MAFLD + MetS 1.22 (1.02–1.47). For CVD, there was a significant increase in HR only in MAFLD + MetS [1.44 (1.18–1.76)]. The results of the analysis stratified by sex showed that MAFLD had a greater impact in men, and MetS had a greater impact in women regarding the development of CAD.

**Conclusions:**

Distinguishing between MetS and/or MAFLD in the presence or absence of type 2 diabetes and according to sex may aid in accurately identifying patients at high risk of cardiovascular disease.

**Supplementary Information:**

The online version contains supplementary material available at 10.1186/s12933-022-01518-4.

## Introduction

The state in which the risk of cardiovascular disease is increased by the accumulation of cardiovascular risk factors such as insulin resistance (IR), glucose intolerance, obesity, hypertension, and dyslipidemia has been long known as metabolic syndrome (MetS) [1]. In recent years, fatty liver disease, including non-alcoholic fatty liver disease (NAFLD), has been gaining attention as a condition with underlying IR similar to MetS [2, 3]. The prevalence of NAFLD has been increasing, particularly in Asia [4]. In addition to IR, in NAFLD as in MetS, there are multiple risk factors such as hypertension, dyslipidemia, and glucose intolerance [5]. Also, as in MetS [6], the major cause of death in patients with NAFLD is cardiovascular disease [7]. However, opinions as to whether NAFLD is a predictor of cardiovascular disease independent of comorbid metabolic abnormalities vary among studies [8, 9]. Furthermore, NAFLD is diagnosed by excluding secondary fatty liver due to significant alcohol consumption or other factors, such as viral liver disease, drug-induced liver injury, etc., while coexisting metabolic abnormalities are not considered in that diagnosis [10]. In 2020, the concept of metabolic dysfunction-associated fatty liver disease (MAFLD) was proposed to obtain an inclusive diagnosis for these patients and recognize the presence of MAFLD (i.e., a metabolic component of liver disease) that can exist despite the presence of other liver disease [11]. The concept of MAFLD more accurately reflects the underlying pathophysiology than the previously used NAFLD and is expected to contribute to improved patient care [12].

It was reported that MAFLD increases the risk of cardiovascular disease as does MetS [13] since the concept of MAFLD is similar to that of MetS except for the presence of fatty liver is essential to its diagnosis [14]. However, no study has examined in the same cohort whether MetS or MAFLD is the more likely to lead to the development of cardiovascular disease. Moreover, we previously reported that MetS was less predictive of cardiovascular disease in diabetic patients than in non-diabetic patients [15]. Similarly, in MAFLD, the presence or absence of type 2 diabetes may have an impact on its predictive ability for cardiovascular disease. It was reported that MAFLD was slightly less predictive of cardiovascular disease in patients with than without type 2 diabetes [13]. In addition, the risk of MAFLD complicated with MetS has not yet been examined. Although there are large sex differences in the prevalence, severity, and risk factors for any of the metabolic abnormalities that are related to fatty liver, cardiovascular disease, and MetS [16], to our knowledge sex differences have not been evaluated in relation to the value of MAFLD to predict cardiovascular disease.

This study aimed to investigate the degree of concordance between the diagnosis of MetS and MAFLD in clinical practice and examine whether a diagnosis of MetS and/or MAFLD would be predictive of cardiovascular disease in Asians using a nationwide claims database and to clarify whether there is a difference between those with and without type 2 diabetes and according to sex.

## Materials and methods

### Study participants

We retrospectively analyzed a large nationwide claims-based database that included claims data for 805,592 employees who purchased health insurance for themselves and their dependents at their companies. Details of the database were reported previously [17, 18]. Persons aged 18 to 72 years who could be monitored for at least 3 years between April 1, 2008 and July 31, 2016 were included in this analysis and when possible were continued to be followed until August 31, 2019. Those with coronary artery disease (CAD) or cerebrovascular disease (CVD) at baseline, with type 1 diabetes, or with missing medical examination data required for analysis, including blood chemistry data required for diagnosis of MetS and MAFLD, were excluded. Ultimately, 570,426 cases (334,401 men and 236,025 women) without a history of either CAD or CVD were included as study participants.

This study was reviewed and approved by the Ethics Committee of Niigata University.

### Definitions

The diagnostic criteria for MetS were based on criteria proposed in a joint statement in 2009 by the International Diabetes Federation (IDF), National Heart, Lung, and Blood Institute, American Heart Association, World Heart Federation, International Atherosclerosis Society, and International Association for the Study of Obesity [1]. MetS was diagnosed based on at least three of the following: 1. Waist circumference (WC) ≥ 90 cm for men and ≥ 80 cm for women, which is the Asian standard proposed by the IDF and World Health Organization, to indicate MetS. 2. Fasting glucose ≥ 5.6 mmol/l (100 mg/dl) or receiving drug treatment for elevated glucose. In addition to the fasting glucose level, hemoglobin A1c (HbA1c) ≥ 38 mmol/mol (5.7%) [11], which is also included in the criteria for MAFLD, was used as a diagnostic criterion for MetS. 3. Triglycerides (TG) ≥ 1.7 mmol/l (150 mg/dl) or receiving drug treatment for elevated TG. 4. Systolic blood pressure (SBP) ≥ 130 and/or diastolic blood pressure (DBP) ≥ 85 mmHg, or receiving antihypertensive drug treatment. 5. High density lipoprotein cholesterol (HDL-C) < 1.0 mmol/l (40 mg/dl) in men, < 1.3 mmol/l (50 mg/dl) in women, or receiving drug treatment for reduced HDL-C.

The fatty liver index (FLI), a widely used clinical indicator of the presence of fatty liver, was used to diagnose fatty liver in this study [13, 19]. An FLI ≥ 60 was indicated as the criterion for the presence of fatty liver in studies of Westerners [19, 20]. Asians were considered to have fatty liver at lower values (e.g., FLI ≥ 30) than Westerners [13]. In a recent large-scale clinical study conducted in South Korea of approximately 3 million people [21], an FLI ≥ 37.09 was an independent cardiovascular disease risk factor regardless of the presence or absence of type 2 diabetes or independent of sex. Therefore, an FLI ≥ 37 was used as the cutoff value in this study. The formula for the FLI is as follows: (e^0.953*loge (TG) + 0.139*BMI + 0.718*loge (gamma−glutamyl transferase: γ−GTP) + 0.053*WC − 15.745^)/(1 + e^0.953*loge(TG) +^ ^0.139*BMI + 0.718*loge (γ−GTP) + 0.053*WC − 15.745^) * 100.

The definition of MAFLD was based on that proposed by the International Consensus Panel in 2020 [11]. Specifically, among participants with an FLI ≥ 37 who (1) had a body mass index (BMI) ≥ 23 (using Asian criteria) and type 2 diabetes or (2) a BMI < 23 without type 2 diabetes, MAFLD was diagnosed based on at least two of the following: WC ≥ 90 cm for men and ≥ 80 cm for women, using Asian criteria; BP ≥ 135/85 mmHg or use of antihypertensive medication; serum TG ≥ 1.7 mmol/l (150 mg/dl) or receiving specific drug treatment; serum HDL-C < 1.0 mmol/l (40 mg/dl) for men and < 1.3 mmol/l (50 mg/dl) for women or receiving specific drug treatment; prediabetes [fasting plasma glucose (FPG) 5.6–6.9 mmol/l (100-125 mg/dl) or HbA1c 38–46 mmol/mol (5.7–6.4%)]; and visceral adiposity index (VAI) ≥ 2.54 [22].

Data on serum high-sensitivity C-reactive protein (CRP) and glucose tolerance were not available in the database used in this study. In addition, since HOMA-IR could not be calculated, VAI was used as an alternative index of IR. VAI was calculated as follows: men [WC/ (39.68 + (1.88 × BMI))] × [TG/1.03] × [1.31/HDL-C] and women [WC/ (36.58 + (1.89 × BMI))] × [TG/0.81] × [1.52/HDL-C] [23].

Type 2 diabetes was diagnosed if FPG ≥ 7.0 mmol/l (126 mg/dl) or HbA1c ≥ 47 mmol/mol (6.5%), or both, or if antidiabetic medication was prescribed regardless of FPG or HbA1c levels. CAD and CVD events were identified using a combination of the diagnostic procedure combination (DPC), International Classification of Diseases, Tenth Revision (ICD-10) codes, prescribed medications, and medical procedures performed. Details are as described previously [24, 25].

### Statistical analysis

Categorical variables were indicated by numbers and percentages, and intergroup comparisons were made by Pearson’s chi-square test. Continuous variables in each group of study participants were classified and tested for normality with the Kolmogorov–Smirnov test to investigate whether a diagnosis of MetS and/or MAFLD would be predictive of cardiovascular disease and whether this varies according to sex or the presence or absence of type 2 diabetes. Since the results did not show a normal distribution, the Kruskal–Wallis test was used for intergroup comparisons, and when significant results were examined by the Bonferroni-Dunn multiple comparisons post hoc test. The impact of MetS and MAFLD to predict the development of CAD and CVD was examined by multivariate Cox proportional hazard analysis. Covariates used were age, sex, current smoking status, low-density lipoprotein cholesterol, and use of statins, which are considered risk factors for cardiovascular events or affect the liver fibrosis [26, 27], in addition to the components of MetS and MAFLD. The presence or absence of type 2 diabetes was used as a covariate in the gender-specific analyses only. Analyses were performed using SPSS (version 28.0, IBM, Chicago, IL, USA). Statistical significance was considered for P < 0.05.

## Results

Table 1 shows the baseline characteristics of study participants according to the presence or absence of MAFLD and/or MetS.

**Table 1:** Baseline characteristics of participants according to the presence or absence of MAFLD or MetS

Characteristics	MAFLD (−), MetS (−) n = 421,922	MAFLD (+), MetS (−) n = 82,033	MAFLD (−), MetS (+) n = 12,457	MAFLD (+), MetS (+) n = 54,014	P value
Sex (men)	217,312 (51.5)	73,484 (89.6)	2103 (16.9)	41,502 (76.8)	< 0.001
Age (years)	44 (40–51)	46 (41–52)	52 (46–59)	48 (42–55)	< 0.001
BMI (kg/m^2^)	21.3 (19.6–23.0)	25.5 (24.1–27.3)	24.2 (22.7–25.7)	28.2 (26.4–30.5)	< 0.001
Waist circumference (cm)	77.2 (72.0–82.0)	88.0 (85.0–93.0)	85.8 (82.5–90.0)	95.5 (92.0–100.6)	< 0.001
Current smoking	95,821 (22.7)	30,973 (37.8)	1467 (11.8)	18,514 (34.3)	< 0.001
HbA1c (mmol/mol)	35 (33–37)	36 (33–38)	39 (37–42)	39 (36–45)	< 0.001
Type 2 diabetes	12,031 (2.9)	7686 (9.4)	1825 (14.7)	15,255 (28.2)	< 0.001
SBP (mmHg)	114 (105–124)	124 (116–132)	132 (122–140)	132 (123–141)	0.001
DBP (mmHg)	70 (64–78)	79 (72–85)	81 (73–88)	84 (77–90)	< 0.001
Pulse pressure (mmHg)	43 (38–50)	45 (40–51)	51 (43–59)	48 (41–56)	< 0.001
Hypertension	43,123 (10.2)	20,429 (24.9)	6039 (48.5)	29,051 (53.8)	< 0.001
Triglycerides (mmol/l)	0.8 (0.6–1.1)	1.6 (1.2–2.3)	1.0 (0.8–1.5)	1.9 (1.4–2.6)	< 0.001
HDL-C (mmo/l)	1.7 (1.4–2.0)	1.3 (1.2–1.6)	1.5 (1.2–1.8)	1.2 (1.1–1.5)	< 0.001
LDL-C (mmol/l)	3.0 (2.5–3.5)	3.4 (2.9–4.0)	3.3 (2.8–3.9)	3.4 (2.9–4.0)	< 0.001
Use of statins	11,068 (2.6)	5008 (6.1)	1623 (13.0)	7003 (13.0)	< 0.001
AST (IU/l)	19 (16–22)	24 (20–29)	19 (16–22)	25 (20–33)	< 0.001
ALT (IU/l)	16 (12–21)	29 (21–42)	17 (13–22)	33 (23–50)	< 0.001
γ-GPT (IU/)	20 (14–30)	53 (35–86)	19 (15–26)	48 (32–77)	< 0.001
Fatty liver index*	8.42 (3.83–17.86)	52.84 (43.95–65.04)	23.82 (15.87–30.69)	72.65 (58.05–85.29)	< 0.001
Visceral adiposity index**	0.72 (0.50–1.06)	1.61 (1.13–2.37)	1.28 (0.83–2.04)	2.22 (1.50–3.25)	< 0.001

Data are presented as median (interquartile range), n (%). Analyses were performed by the Kruskal–Wallis test or Pearson’s chi-square test across groups

MAFLD: metabolic dysfunction-associated fatty liver disease; MetS: metabolic syndrome; BMI: mass index; SBP, systolic blood pressure; DBP: diastolic blood pressure; HDL-C: high-density lipoprotein cholesterol; LDL-C: low-density lipoprotein cholesterol; gamma-glutamyl transferase: γ-GTP; WC: waist circumference

^*^Fatty liver index: (e ^0.953*loge (TG) + 0.139*BMI + 0.718*loge (γ−GTP) + 0.053*WC − 15.745^)/(1 + e ^0.953*loge(TG) + ^^0.139*BMI + 0.718*loge (γ−GTP) + 0.053*WC − 15.745^) * 100

^**^Visceral adiposity index: Women; [WC/(36.58 + (1.89 × BMI))] × [TG/0.81] × [1.52/HDL-C], Men; [WC/(39.68 + (1.88 × BMI))] × [TG/1.03] × [1.31/HDL-C] WC: waist circumference. TG: triglycerides

**Fig. 1:**
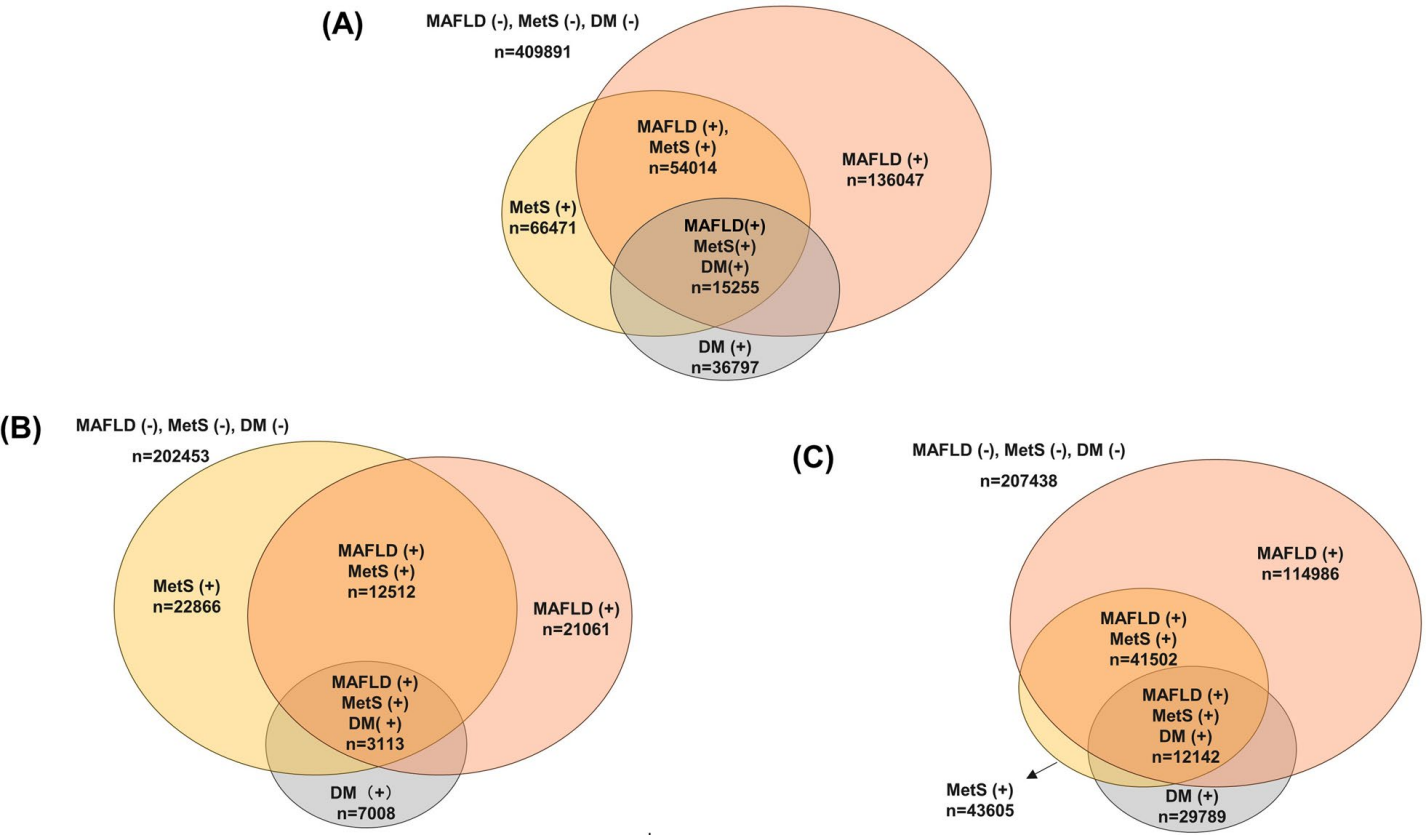
Distribution of participants according to the presence or absence of metabolic dysfunction-associated fatty liver disease (MAFLD), metabolic syndrome (MetS), and type 2 diabetes (DM). **A** Distribution of total participant population according to the presence or absence of MAFLD, MetS, and DM. **B** Distribution of women according to the presence or absence of MAFLD, MetS, and DM. **C** Distribution of men according to the presence or absence of MAFLD, MetS, and DM

The median follow-up period was 5.2 years, and the prevalence of type 2 diabetes among all participants was about 6.5%. In the MAFLD only group (without MetS) indices reflecting IR such as VAI were significantly higher than in the MetS-only (without MAFLD) group as was the proportion of smokers. On the other hand, the MetS-only group had a higher proportion of older patients and patients with hypertension and type 2 diabetes as well as significantly higher rates of the use of statins and significantly higher systolic blood pressure (SBP) and pulse pressure values compared to the MAFLD-only group. The above results in the comparison between the MAFLD only group and the MetS only group were similar whether they were compared only between non-diabetic participants or between diabetic participants (Additional file 1: Tables S1, S2). They were also similar when compared by sex (Additional file 1: Tables S3, S4). In addition, the distribution and prevalence of MetS and MAFLD differed greatly between men and women (Fig. 1). Specifically, in women, prevalences of MetS and MAFLD were similar, and the coexistence of MetS and MAFLD was not high. On the other hand, in men, the prevalence of MAFLD was much higher than in women**,** and male patients with the diagnosis of MetS had a more than 80% chance of also having MAFLD. At the same time, the proportion of MAFLD patients also fulfilling the diagnosis of MetS was not high.

**Fig. 2:**
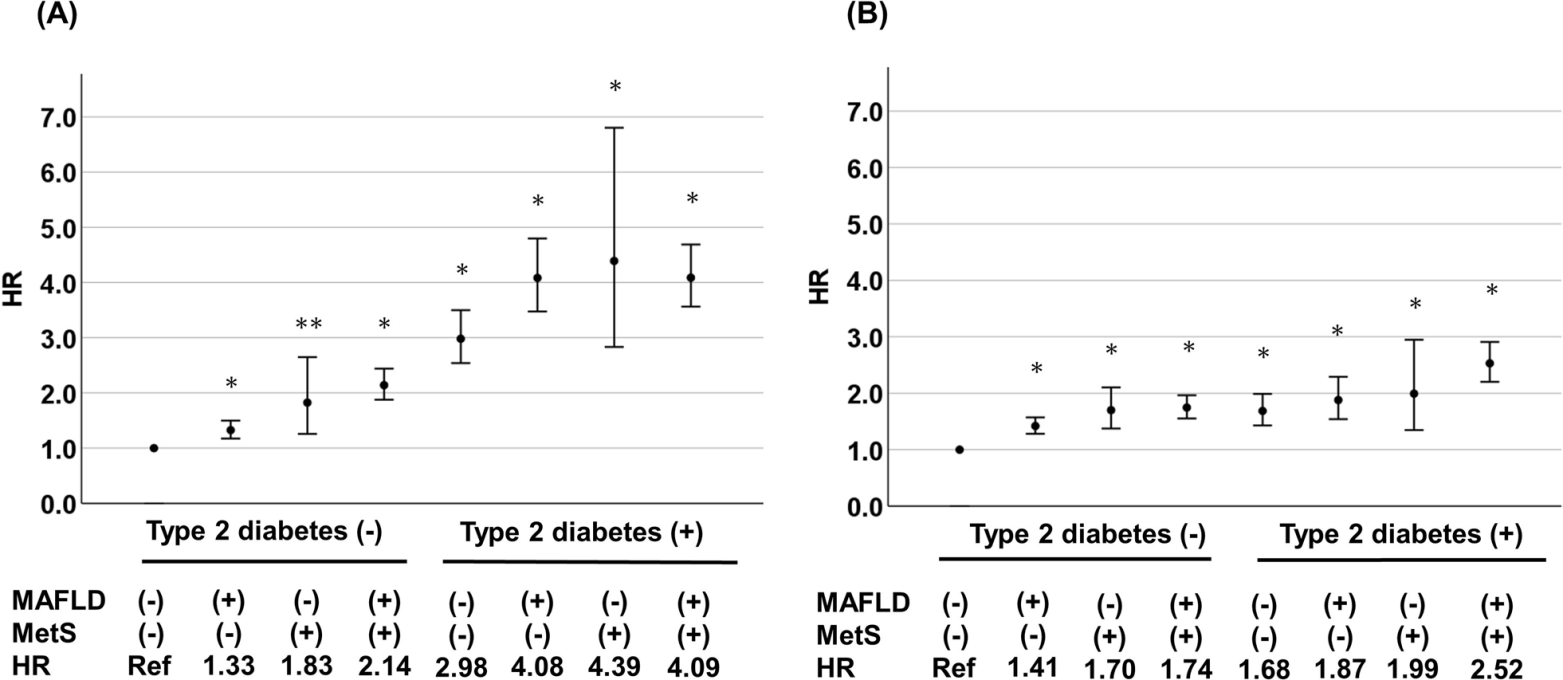
Impact of metabolic dysfunction-associated fatty liver disease (MAFLD), metabolic syndrome (MetS), and type 2 diabetes (DM) on cardiovascular disease. **A** Impact of MAFLD, MetS, and DM on coronary artery disease (CAD). **B** Impact of MAFLD, MetS, and DM on cerebrovascular disease (CVD). Analysis was performed using a Cox proportional hazards model adjusted for age, sex, current smoking, LDL-C and use of statin. *P < 0.001 vs. Group with neither MAFLD, MetS nor DM. **P < 0.01 vs. Group with neither MAFLD, MetS nor DM. MAFLD, metabolic dysfunction-associated fatty liver disease; MetS, metabolic syndrome; DM, type 2 diabetes; HR, hazard ratio

The results of analyses of the impact of MAFLD and MetS on the incidence of CAD and CVD in all participants and on participants when stratified by the presence or absence of type 2 diabetes using the Cox proportional hazard model are shown in Fig. 2 and Table 2. In the analysis of overall participants, the risk of developing CAD [multivariable-adjusted hazard ratio (HR)] was significantly increased in the group with either MAFLD only or MetS only compared with groups having neither MAFLD, MetS, nor type 2 diabetes. There was a further increase in the HR for CAD with the coexistence of both MAFLD and MetS, but it was not as high as the HR with type 2 diabetes only (Fig. 2). The risk of developing CVD was significantly increased in the MAFLD-only and MetS-only groups, and the HRs for MetS and MetS + MAFLD were similar to that for type 2 diabetes only (Fig. 2). In addition, the HRs for MetS was slightly higher than that for MAFLD for both CAD and CVD.

**Table 2:** CAD and CVD risk according to presence or absence of MAFLD or MetS in participants stratified according to with or without type 2 diabetes

Group		Events	Rate*	HR (95% confidence interval)							
				Model 1				Model 2			
		CAD/CVD	CAD/CVD	CAD	P-value	CVD	P-value	CAD	P-value	CVD	P-value
Total		2252/3128	0.71/0.98								
	Neither MAFLD nor MetS	1002/1770	0.42/0.75	1.00 (reference)		1.00 (reference)		1.00 (reference)		1.00 (reference)	
	MAFLD only	578/633	1.27/1.39	**1.78 (1.61–1.98)**	< 0.001	**1.45 (1.32–1.59)**	< 0.001	**1.47 (1.33–1.63)**	< 0.001	**1.40 (1.28–1.54)**	< 0.001
	Mets only	51/121	0.75/1.79	**2.23 (1.67–2.97)**	< 0.001	**1.71 (1.42–2.06)**	< 0.001	**2.02 (1.51–2.70)**	< 0.001	**1.67 (1.38–2.02)**	< 0.001
	Both MAFLD and MetS	621/604	2.10/2.04	**2.98 (2.69–3.29)**	< 0.001	**1.98 (1.80–2.17)**	< 0.001	**2.33 (2.11–2.58)**	< 0.001	**1.89 (1.71–2.08)**	< 0.001
Without type 2 diabetes	Total	1540/2581	0.52/0.87								
	Neither MAFLD nor MetS	803/1606	0.35/0.70	1.00 (reference)		1.00 (reference)		1.00 (reference)		1.00 (reference)	
	MAFLD only	386/524	0.94/1.27	**1.59 (1.41–1.80)**	< 0.001	**1.45 (1.31–1.61)**	< 0.001	**1.32 (1.17–1.50)**	< 0.001	**1.41 (1.28–1.57)**	< 0.001
	MetS only	30/95	0.52/1.64	**1.97 (1.36–2.85)**	< 0.001	**1.69 (1.37–2.09)**	< 0.001	**1.78 (1.22–2.58)**	0.003	**1.66 (1.34–2.06)**	< 0.001
	Both MAFLD and MetS	321/356	1.51/1.68	**2.64 (2.32–3.00)**	< 0.001	**1.80 (1.60–2.02)**	< 0.001	**2.10 (1.84–2.39)**	< 0.001	**1.73 (1.54–1.95)**	< 0.001
With type 2 diabetes	Total	712/547	3.54/2.71								
	Neither MAFLD nor MetS	199/164	3.01/2.47	1.00 (reference)		1.00 (reference)		1.00 (reference)		1.00 (reference)	
	MAFLD only	192/109	4.52/2.55	**1.46 (1.20–1.79)**	< 0.001	1.10 (0.86–1.41)	0.430	**1.29 (1.06–1.58)**	0.013	1.08 (0.84–1.38)	0.547
	MetS only	21/26	2.20/2.72	1.28 (0.81–2.04)	0.294	1.23 (0.80–1.90)	0.355	1.34 (0.84–2.13)	0.224	1.24 (0.80–1.92)	0.334
	Both MAFLD and MetS	300/248	3.61/2.98	**1.38 (1.15–1.66)**	< 0.001	**1.45 (1.19–1.77)**	< 0.001	**1.22 (1.02–1.47)**	0.034	**1.44 (1.18–1.76)**	< 0.001

Analysis was performed using a Cox proportional hazard model. Values in bold are statistically significant (p < 0.05).

Model 1: Adjusted for age and sex. Model 2: Adjusted for Model 1 plus current smoking, LDL-C and use of statin

CAD: coronary artery disease; CVD: cerebrovascular disease; MAFLD: metabolic dysfunction-associated fatty liver disease; MetS: metabolic syndrome; HR: hazard ratio; MAFLD only: MAFLD without MetS; MetS only: MetS without MAFLD. Rate* per 1000 person-years

In the analysis of the subpopulation classified by the presence or absence of type 2 diabetes, the trend for both CAD and CVD in the group without type 2 diabetes was similar to that in the overall analysis (Table 2). However, in the type 2 diabetes group, there was no significant increase in the risk of developing CAD with MetS only but there was a significant increase in risk with MAFLD only. On the other hand, with neither MAFLD only nor MetS only was there a significant increase in the risk of CVD, but such an increase was evident only when these conditions coexisted.

The results of the analysis stratified by sex are shown in Table 3. In men, MetS only did not increase the risk of CAD, although MAFLD only (and MAFLD + MetS) did. In contrast, in women MAFLD only did not increase the risk of CAD, but MetS only (and MetS + MAFLD) did. On the other hand, there was a significant increase in the risk of CVD in both men and women for either MAFLD or MetS only, with a slightly greater increase in risk for MetS only than for MAFLD only in both men and women.

**Table 3:** Sex differences in CAD or CVD risk according to the presence or absence of MAFLD or MetS

Group		Events	Rate*	HR (95% confidence interval)					
				Model 1		Model 2		Model 3	
					P-value		P-value		P-value
CAD Men	Total	2108	1.14						
	Neither MAFLD nor MetS	929	0.78	1.00 (reference)		1.00 (reference)		1.00 (reference)	
	MAFLD only	576	1.40	**1.74 (1.57–1.93)**	< 0.001	**1.45 (1.31–1.61)**	< 0.001	**1.35 (1.21–1.50)**	< 0.001
	MetS only	23	2.07	**1.61 (1.06–2.44)**	0.024	**1.61 (1.06–2.43)**	0.025	1.34 (0.88–2.03)	0.170
	Both MAFLD and MetS	580	2.55	**2.84 (2.56–3.15)**	< 0.001	**2.24 (2.02–2.49)**	< 0.001	**1.72 (1.54–1.93)**	< 0.001
Women	Total	144	0.12						
	Neither MAFLD nor MetS	68	0.06	1.00 (reference)		1.00 (reference)		1.00 (reference)	
	MAFLD only	7	0.15	2.03 (0.93–4.42)	0.075	1.63 (0.75–3.57)	0.220	1.48 (0.68–3.24)	0.322
	MetS only	28	0.49	**3.56 (2.25–5.64)**	< 0.001	**3.15 (1.98–4.99)**	< 0.001	**2.64 (1.65–4.23)**	< 0.001
	Both MAFLD and MetS	41	0.60	**5.63 (3.78–8.39)**	< 0.001	**4.39 (2.91–6.61)**	< 0.001	**3.08 (1.97–4.82)**	< 0.001
CVD Men	Total	2277	1.23						
	Neither MAFLD nor MetS	1170	0.97	1.00 (reference)		1.00 (reference)		1.00 (reference)	
	MAFLD only	590	1.45	**1.43 (1.30–1.58)**	< 0.001	**1.40 (1.26–1.54)**	< 0.001	**1.36 (1.23–1.50)**	< 0.001
	MetS only	31	2.79	**1.80 (1.26–2.58)**	0.001	**1.81 (1.27–2.59)**	0.001	**1.68 (1.18–2.41)**	0.004
	Both MAFLD and MetS	486	2.13	**1.91 (1.72–2.12)**	< 0.001	**1.83 (1.64–2.04)**	< 0.001	**1.65 (1.48–1.85)**	< 0.001
Women	Total	851	0.64						
	Neither MAFLD nor MetS	600	0.52	1.00 (reference)		1.00 (reference)		1.00 (reference)	
	MAFLD only	43	0.92	**1.53 (1.12–2.08)**	0.008	**1.43 (1.05–1.95)**	0.025	**1.40 (1.03–1.92)**	0.033
	MetS only	90	1.59	**1.78 (1.41–2.23)**	< 0.001	**1.72 (1.37–2.17)**	< 0.001	**1.66 (1.31–2.10)**	< 0.001
	Both MAFLD and MetS	118	1.74	**2.30 (1.88–2.82)**	< 0.001	**2.12 (1.72–2.61)**	< 0.001	**1.96 (1.57–2.45)**	< 0.001

Analysis was performed using a Cox proportional hazard model. Model 1: Adjusted for age. Model 2: Adjusted for Model 1 plus current smoking, LDL-C and use of statin. Model 3: Adjusted for Model 2 plus presence of type 2 diabetes. Values in bold are statistically significant (p<0.05). CAD: coronary artery disease; CVD: cerebrovascular disease; MAFLD: metabolic dysfunction-associated fatty liver disease; MetS: metabolic syndrome; HR: hazard ratio; MAFLD only: MAFLD without MetS; MetS only: MetS without MAFLD. Rate* per 1000 person-years

## Discussion

This historical cohort study aimed to clarify the association between the presence of MetS and MAFLD, which share the common pathophysiological background of IR, and the risk of developing cardiovascular disease. Results showed that, overall, MetS had slightly superior predictive ability for the development of cardiovascular disease than MAFLD. However, the results differed greatly depending on sex and the presence or absence of type 2 diabetes as well as between CAD and CVD.

As in our previous report, MetS as a predictor of cardiovascular disease was reduced in type 2 diabetes patients who were already at high risk for atherosclerotic disease [15]. However, in predicting the development of CAD but not CVD, MAFLD was shown to be useful even in patients with type 2 diabetes in our study. MAFLD was associated with more severe IR as indicated by higher values for VAI, BMI, WC, and FLI than for MetS only. This result can be interpreted as indicating that the presence of more severe hyperinsulinemia is an additional risk factor for CAD, even in those with type 2 diabetes.

Neither the presence of MetS only or MAFLD only was useful in predicting the development of CVD in persons with type 2 diabetes. However, if both were present, the CVD risk increased even in persons with type 2 diabetes. The severity of fatty liver and the progression of liver fibrosis have been significantly correlated with the risk of stroke [28]. In this study, the FLI was significantly higher in the group with both MAFLD and MetS compared with the other groups, even in groups with diabetic patients. An increased FLI reflects the progression of liver fibrosis to some extent [29].

Patients with type 2 diabetes are more likely to develop advanced MAFLD (steatohepatitis, fibrosis, cirrhosis, etc.) than those without type 2 diabetes [30, 31]. Also, there is a close bidirectional relationship between cardiovascular disease and advanced MAFLD [32, 33]. MAFLD may cause difficulty in achieving adequate glycemic control in patient with diabetes [34]. Indeed, in the current study the presence of MetS only (without MAFLD) did not significantly increase the risk of CAD in people with type 2 diabetes. Those findings imply that clinicians should pay close attention to persons with MAFLD and type 2 diabetes as being at high risk for cardiovascular disease [33].

As to sex differences in the impact of MetS and MAFLD on the development of cardiovascular disease, MetS had a strong impact on the development of CAD in women. Even when women have risk factors for CAD, premenopausal women are less likely to develop fatty liver. Estrogen has been reported to have a strong protective effect against fatty liver in premenopausal women [16, 35, 36]. Unfortunately, information on menopause was not available for this study, but considering that 72% of female participants were younger than 50 years it can be inferred that the majority of female participants were premenopausal.

MAFLD did have a strong impact on the development of CAD in men. In men, unlike in women, MetS was almost always combined with MAFLD, and a MetS diagnosis captured only a small proportion of those having concomitant MAFLD. Thus, many patients with MAFLD are overlooked only by a diagnosis of MetS. Thus, for assessment of risk of CAD, in men it is more appropriate to use a diagnosis of MAFLD. In addition to the influence of sex hormones, a wide variety of mechanisms were reported to be responsible for these sex differences in both men and women [37, 38]. Further research is expected to elucidate these mechanisms.

A strength of the current study is that it is the first large-scale clinical study to directly compare the impact of MetS and MAFLD on the risk of developing cardiovascular disease, stratified by the presence or absence of type 2 diabetes and by sex, using real-world data. It is known that the impact of MetS and MAFLD on cardiovascular events is strongly influenced by the presence or absence of type 2 diabetes and by sex [16, 39], but there are insufficient detailed studies stratified by these factors [16]. The combination of DPC, ICD-10 codes, contents of prescribed medications, and medical procedures performed enabled accurate identification of CAD and CVD events [24, 25].

This study had the following limitations. First, we used the FLI in the identification of fatty liver, because no imaging or histopathological information was available. However, the FLI has been considered appropriate and is widely used to identify fatty liver in large-scale clinical studies [11, 13]. In addition, the currently proposed diagnostic criteria for MAFLD allowed the use of a biomarker-based index such as the FLI to identify fatty liver [11]. Nevertheless, it is difficult to distinguish which components of the FLI (BMI, WC, TG levels and γ-GPT levels [40]) or hepatic fat that were responsible for the increased risk. Thus, our findings should be confirmed with imaging or histopathological information. Second, since the database used is for Japanese company employees and their dependents, few elderly people were included. Also, no information on menopause was available, which may have played a role in the results. Third, the diagnostic definition of MAFLD, unlike NAFLD, includes either the presence or absence of liver diseases, and the impact of liver diseases such as viral hepatitis could not be considered due to lack of information. Fourth, data on high-sensitivity CRP, HOMA-IR, and the glucose tolerance test, which are diagnostic criteria for MAFLD, were not available in this database, making it possible that MAFLD was underdiagnosed. However, we calculated and used VAI as an alternative index for HOMA-IR, which is considered to reflect IR without being inferior to HOMA-IR [22]. Fifth, liver fibrosis is associated with the development of cardiovascular disease [41], but the database had no information on liver fibrosis (including platelet count). Finally, although we considered a large number of risk factors that might have influenced the development of cardiovascular disease, we did not have information on renal function, atrial fibrillation, etc., that may present a risk of cardiovascular disease.

## Conclusion

Although MAFLD and MetS are closely related and refer to very similar pathological conditions, their coexistence is not necessarily high. In addition, when the diagnoses of MetS and MAFLD are considered to be predictive of cardiovascular disease, both are useful, but their usefulness differs depending on sex and the presence or absence of type 2 diabetes. The use of the respective diagnoses of MetS and MAFLD for this purpose should account for sex and the presence or absence of type 2 diabetes. This strategy may help to accurately identify patients at risk of developing cardiovascular disease and to prevent its progression through early and active interventions. It is essential to conduct further studies aimed at investigating the differences in pathophysiology in each patient group when using the presence, absence, or combination of MetS and MAFLD as predictors of cardiovascular disease.

## Supplementary Information

**Additional file 1:** **Table S1.** Baseline characteristics of participants without type 2 diabetes according to the classification by the presence or absence of MAFLD or Mets. **Table S2.** Baseline characteristics of participants with type 2 diabetes according to the classification by the presence or absence of MAFLD or Mets. **Table S3.** Baseline characteristics of women classified by the presence or absence of MAFLD or MetS. **Table S4.** Baseline characteristics of men classified by the presence or absence of MAFLD or MetS.

## Data Availability

Restrictions apply to the availability of some or all data generated or analyzed during this study to preserve patient confidentiality or because they were used under license. The corresponding author will on request detail the restrictions and any conditions under which access to some data may be provided.

## Abbreviations

MetS: Metabolic syndrome
MAFLD: Metabolic dysfunction associated fatty liver disease
CAD: Coronary artery disease
CVD: Cerebrovascular disease
IR: Insulin resistance
NAFLD: Non-alcoholic fatty liver disease
WC: Waist circumference
TG: Triglycerides
SBP: Systolic blood pressure
DBP: Diastolic blood pressure
HDL-C: HDL cholesterol
LDL-C: LDL cholesterol
γ-GTP: Gamma-glutamyl transferase
FLI: Fatty liver index
FPG: Fasting plasma glucose
VAI: Visceral adiposity index
CRP: C-reactive protein
DPC: Diagnostic procedure combination
ICD-10: International classification of disease, tenth revision
