# Supplementary material for: Impact of metabolic syndrome and metabolic dysfunction-associated fatty liver disease on cardiovascular risk by the presence or absence of type 2 diabetes and according to sex
Source: Cardiovasc Diabetol. 2022 Jun 2;21:90. doi: 10.1186/s12933-022-01518-4 (PMC9161475; doi:10.1186/s12933-022-01518-4)
Supplement: Supplementary file 1 — Additional file 1: Table S1. Baseline characteristics of participants without type 2 diabetes according to the classification by the presence or absence of MAFLD or Mets. Table S2. Baseline characteristics of participants with type 2 diabetes according to the classification by the presence or absence of MAFLD or Mets. Table S3. Baseline characteristics of women classified by the presence or absence of MAFLD or MetS. Table S4. Baseline characteristics of men classified by the presence or absence of MAFLD or MetS. [file 12933_2022_1518_MOESM1_ESM.docx]

| Characteristics |  | MAFLD (-), Mets (-)  n = 409891 | MAFLD (+), Mets (-)  n = 74347 | MAFLD (-), Mets (+)  n = 10632 | MAFLD (+), Mets (+)  n = 38759 | P value |
| --- | --- | --- | --- | --- | --- | --- |
| Sex (men) |  | 207438 (50.6) | 66251 (89.1) | 1563 (14.7) | 29360 (75.8) | <0.001 |
| Age (years) | | 44 (39-50) | 46 (40-52) | 52 (45-58) | 47 (42-54) | <0.001 |
| BMI (kg/m^2^) | | 21.3 (19.6-23.0) | 25.6 (24.1-27.4) | 24.1 (22.6-25.5) | 27.9 (26.2-30.0) | <0.001 |
| Waist circumference (cm) | | 77.0 (72.0-82.0) | 88.2 (85.0-93.0) | 85.5 (82.5-90.0) | 95.0 (91.7-99.5) | <0.001 |
| Current smoking |  | 91706 (22.4) | 27658 (37.2) | 1205 (11.3) | 13085 (33.8) | <0.001 |
| HbA1c (mmol/mol) | | 35 (33-37) | 36 (33-37) | 38 (36-40) | 38 (35-40) | <0.001 |
| SBP (mmHg) | | 114 (104-124) | 124 (115-132) | 132 (122-140) | 132 (122-140) | <0.001 |
| DBP (mmHg) | | 70 (63-78) | 78 (72-85) | 81 (73-88) | 84 (77-90) | <0.001 |
| Pulse pressure (mmHg) | | 43 (38-49) | 45 (40-51) | 51 (43-58) | 48 (42-54) | <0.001 |
| Hypertension |  | 39348 (9.6) | 17672 (23.8) | 4870 (45.8) | 19013 (49.1) | <0.001 |
| Triglycerides (mmol/l) | | 0.8 (0.6-1.1) | 1.6 (1.2-2.3) | 1.1 (0.8-1.5) | 1.9 (1.4-2.6) | <0.001 |
| HDL-C (mmol/l) | | 1.7 (1.5-2.0) | 1.3 (1.2-1.6) | 1.5 (1.2-1.8) | 1.2 (1.1-1.5) | <0.001 |
| LDL-C (mmol/l) | | 3.0 (2.5-3.5) | 3.4 (2.9-4.0) | 3.4 (2.9-3.9) | 3.5 (3.0-4.0) | <0.001 |
| Use of statins |  | 8892 (2.2) | 3634 (4.9) | 1623 (13.0) | 3376 (8.7) | <0.001 |
| AST (IU/L) | | 19 (16-22) | 24 (20-29) | 19 (16-22) | 24 (20-31) | <0.001 |
| ALT (IU/L) | | 16 (12-21) | 29 (21-41) | 16 (13-22) | 32 (22-48) | <0.001 |
| γ-GPT (IU/L) | | 20 (14-29) | 52 (34-85) | 19 (15-26) | 47 (31-75) | <0.001 |
| Fatty liver index* | | 8.22 (3.77-17.49) | 52.61 (43.84-64.69) | 23.31 (15.49-30.42) | 70.91 (56.72-83.44) | <0.001 |
| Visceral adiposity index** | | 0.71 (0.50-1.05) | 1.60 (1.12-2.35) | 1.31 (0.85-2.11) | 2.26 (1.55-3.27) | <0.001 |

**Supplemental table 1.** Baseline characteristics of participants without type 2 diabetes according to the classification by the presence or absence of MAFLD or Mets.

Data are presented as median (interquartile range), n (%). Analyses were performed by the Kruskal-Wallis test or Pearson’s chi-square test across groups.

MAFLD, metabolic dysfunction-associated fatty liver disease; MetS, metabolic syndrome; BMI, mass index; SBP, systolic blood pressure; DBP,: diastolic blood pressure; HDL-C, high-density lipoprotein cholesterol; LDL-C, low-density lipoprotein cholesterol; gamma-glutamyl transferase, γ-GTP; WC, waist circumference.

*: Fatty liver index: (e ^0.953*loge (TG) + 0.139*BMI + 0.718*loge (γ-GTP) + 0.053*WC - 15.745^) / (1 + e ^0.953*loge (TG) + 0.139*BMI + 0.718*loge (γ-GTP) + 0.053*WC - 15.745^) * 100

**: Visceral adiposity index: Women; [WC/ (36.58 + (1.89 × BMI))] × [TG/0.81] × [1.52/HDL-C], Men; [WC/ (39.68 + (1.88 × BMI))] × [TG/1.03] × [1.31/HDL-C] WC: waist circumference. TG: triglycerides.

**Supplemental table 2.** Baseline characteristics of participants with type 2 diabetes according to the classification by the presence or absence of MAFLD or Mets.

| Characteristics |  | MAFLD (-), Mets (-)  n = 12031 | MAFLD (+), Mets (-)  n = 7686 | MAFLD (-), Mets (+)  n = 1825 | MAFLD (+), Mets (+)  n = 15255 | P value |
| --- | --- | --- | --- | --- | --- | --- |
| Sex (men) |  | 9874 (82.1) | 7233 (94.1) | 540 (29.6) | 12142 (79.6) | <0.001 |
| Age (years) | | 54 (48-60) | 52 (46-57) | 56 (50-61) | 51 (45-56) | <0.001 |
| BMI (kg/m^2^) | | 22.4 (20.8-23.9) | 25.2 (23.7-26.8) | 24.8 (23.3-26.3) | 29.0 (27.0-31.8) | <0.001 |
| Waist circumference (cm) | | 81.0 (76.5-84.5) | 87.1 (84.2-89.5) | 88.0 (84.0-91.5) | 97.5 (93.0-104.0) | <0.001 |
| Current smoking |  | 4115 (34.2) | 3315 (43.1) | 282 (14.4) | 5429 (35.6) | <0.001 |
| HbA1c (mmol/mol) | | 49 (44-56) | 50 (46-59) | 49 (45-56) | 51 (47-61) | <0.001 |
| SBP (mmHg) | | 123 (113-134) | 126 (118-136) | 133 (124-142) | 134 (124-144) | <0.001 |
| DBP (mmHg) | | 76 (69-83) | 80 (73-86) | 80 (72-87) | 84 (77-90) | <0.001 |
| Pulse pressure (mmHg) | | 47 (40-54) | 47 (41-54) | 53 (46-61) | 50 (43-58) | <0.001 |
| Hypertension |  | 3775 (31.4) | 2757 (35.9) | 1169 (64.1) | 10038 (65.8) | <0.001 |
| Triglycerides (mmol/l) | | 1.0 (0.7-1.3) | 1.7 (1.3-2.6) | 1.0 (0.7-1.3) | 1.8 (1.3-2.6) | <0.001 |
| HDL-C (mmol/l) | | 1.5 (1.3-1.8) | 1.3 (1.1-1.6) | 1.5 (1.3-1.8) | 1.2 (1.1-1.5) | <0.001 |
| LDL-C (mmol/l) | | 3.1 (2.6-3.6) | 3.3 (2.7-3.9) | 3.1 (2.6-3.7) | 3.3 (2.8-3.9) | <0.001 |
| Use of statins |  | 2176 (18.1) | 1374 (17.9) | 562 (30.8) | 3627 (23.8) | <0.001 |
| AST (IU/L) | | 19 (16-24) | 25 (20-33) | 20 (17-24) | 26 (20-37) | <0.001 |
| ALT (IU/L) | | 19 (15-26) | 32 (22-47) | 19 (15-26) | 36 (24-56) | <0.001 |
| γ-GPT (IU/L) | | 26 (19-38) | 61 (40-101) | 21 (16-28) | 51 (34-82) | <0.001 |
| Fatty liver index* | | 17.21 (9.18-26.30) | 55.29 (45.14-68.26) | 26.69 (19.04-31.98) | 77.48 (61.97-89.44) | <0.001 |
| Visceral adiposity index** | | 0.87 (0.57-1.28) | 1.72 (1.20-2.73) | 1.10 (0.76-1.70) | 2.09 (1.38-3.19) | <0.001 |

Data are presented as median (interquartile range), n (%). Analyses were performed by the Kruskal-Wallis test or Pearson’s chi-square test across groups.

MAFLD, metabolic dysfunction-associated fatty liver disease; MetS, metabolic syndrome; BMI, mass index; SBP, systolic blood pressure; DBP,: diastolic blood pressure; HDL-C, high-density lipoprotein cholesterol; LDL-C, low-density lipoprotein cholesterol; gamma-glutamyl transferase, γ-GTP; WC, waist circumference.

*: Fatty liver index: (e ^0.953*loge (TG) + 0.139*BMI + 0.718*loge (γ-GTP) + 0.053*WC - 15.745^) / (1 + e ^0.953*loge (TG) + 0.139*BMI + 0.718*loge (γ-GTP) + 0.053*WC - 15.745^) * 100

**: Visceral adiposity index: Women; [WC/ (36.58 + (1.89 × BMI))] × [TG/0.81] × [1.52/HDL-C], Men; [WC/ (39.68 + (1.88 × BMI))] × [TG/1.03] × [1.31/HDL-C] WC: waist circumference. TG: triglycerides.

| Characteristics |  | MAFLD (-), MetS (-)  n = 204610 | MAFLD (+), MetS (-)  n = 8549 | MAFLD (-), MetS(+)  n = 10354 | MAFLD (+), MetS (+)  n = 12512 | P value |
| --- | --- | --- | --- | --- | --- | --- |
| Age (years) | | 43 (39-49) | 46 (40-52) | 52 (45-58) | 49 (43-56) | <0.001 |
| BMI (kg/m^2^) | | 20.4 (18.9-22.2) | 28.1 (26.0-30.4) | 23.8 (22.4-25.3) | 28.4 (26.2-31.2) | <0.001 |
| Waist circumference (cm) | | 74.5 (69.8-79.5) | 94.0 (89.0-99.0) | 84.5 (82.0-87.9) | 94.0 (89.0-100.0) | <0.001 |
| Current smoking |  | 16752 (8.2) | 1071 (12.5) | 840 (8.1) | 1674 (13.4) | <0.001 |
| HbA1c (mmol/mol) | | 35 (33-37) | 36 (34-38) | 39 (37-42) | 39 (37-45) | <0.001 |
| Type 2 diabetes |  | 2157 (1.1) | 453 (5.3) | 1285 (12.4) | 3113 (24.9) | <0.001 |
| SBP (mmHg) | | 110 (101-120) | 121 (112-128) | 132 (122-140) | 133 (122-143) | <0.001 |
| DBP (mmHg) | | 67 (61-74) | 75 (68-81) | 80 (72-87) | 81 (74-88) | <0.001 |
| Pulse pressure (mmHg) | | 42 (37-49) | 46 (40-52) | 52 (44-59) | 51 (44-59) | <0.001 |
| Hypertension |  | 12299 (6.0) | 1244 (14.6) | 4721 (45.6) | 6235 (49.8) | <0.001 |
| Triglycerides (mmol/l) | | 0.7 (0.5-0.9) | 1.2 (1.0-1.5) | 1.1 (0.8-1.6) | 1.8 (1.3-2.3) | <0.001 |
| HDL-C (mmol/l) | | 1.9 (1.6-2.1) | 1.6 (1.4-1.8) | 1.5 (1.2-1.8) | 1.3 (1.2-1.6) | <0.001 |
| LDL-C (mmol/l) | | 2.9 (2.4-3.4) | 3.4 (2.9-4.1) | 3.4 (2.9-4.0) | 3.6 (3.0-4.2) | <0.001 |
| Use of statins |  | 4272 (2.1) | 491 (5.7) | 1342 (13.0) | 1750 (14.0) | <0.001 |
| AST (IU/L) | | 17 (15-20) | 20 (17-26) | 19 (16-22) | 22 (18-29) | <0.001 |
| ALT (IU/L) | | 13 (10-17) | 21 (16-32) | 16 (13-21) | 25 (18-39) | <0.001 |
| γ-GPT (IU/L) | | 15 (12-20) | 31 (21-55) | 19 (14-25) | 33 (23-52) | <0.001 |
| Fatty liver index* | | 4.56 (2.53-9.13) | 50.11 (42.48-63.00) | 21.90 (14.55-29.10) | 61.55 (48.39-77.57) | <0.001 |
| Visceral adiposity index** | | 0.69 (0.49-0.99) | 1.50 (1.11-1.98) | 1.42 (0.92-2.22) | 2.49 (1.70-3.56) | <0.001 |

**Supplemental table 3.**  Baseline characteristics of women classified by the presence or absence of MAFLD or MetS.

Data are presented as median (interquartile range), n (%). Analyses were performed by the Kruskal-Wallis test or Pearson’s chi-square test across groups.

MAFLD, metabolic dysfunction-associated fatty liver disease; MetS, metabolic syndrome; BMI, mass index; SBP, systolic blood pressure; DBP,: diastolic blood pressure; HDL-C, high-density lipoprotein cholesterol; LDL-C, low-density lipoprotein cholesterol; gamma-glutamyl transferase, γ-GTP; WC, waist circumference.

*: Fatty liver index: (e ^0.953*loge (TG) + 0.139*BMI + 0.718*loge (γ-GTP) + 0.053*WC - 15.745^) / (1 + e ^0.953*loge (TG) + 0.139*BMI + 0.718*loge (γ-GTP) + 0.053*WC - 15.745^) * 100

**: Visceral adiposity index: Women; [WC/ (36.58 + (1.89 × BMI))] × [TG/0.81] × [1.52/HDL-C], Men; [WC/ (39.68 + (1.88 × BMI))] × [TG/1.03] × [1.31/HDL-C] WC: waist circumference. TG: triglycerides.

| Characteristics |  | MAFLD (-), MetS (-)  n = 217312 | MAFLD (+), MetS (-)  n = 73484 | MAFLD (-), MetS (+)  n = 2103 | MAFLD (+), MetS (+)  n = 41502 | P value |
| --- | --- | --- | --- | --- | --- | --- |
| Age (years) | | 45 (40-52) | 46 (41-53) | 53 (47-59) | 48 (42-54) | <0.001 |
| BMI (kg/m^2^) | | 22.1 (20.6-23.5) | 25.3 (24.0-27.0) | 25.5 (24.4-26.5) | 28.1 (26.5-30.3) | <0.001 |
| Waist circumference (cm) | | 79.6 (75.0-83.5) | 88.0 (84.5-92.0) | 91.6 (90.5-93.4) | 96.0 (92.5-101.0) | <0.001 |
| Current smoking |  | 79069 (36.4) | 29902 (40.7) | 627 (29.8) | 16840 (40.6) | <0.001 |
| HbA1c (mmol/mol) | | 35 (33-37) | 36 (33-38) | 39 (36-43) | 39 (36-45) | <0.001 |
| Type 2 diabetes |  | 9874 (4.5) | 7233 (9.8) | 540 (25.7) | 12142 (29.3) | <0.001 |
| SBP (mmHg) | | 118 (109-127) | 124 (116-133) | 133 (125-140) | 132 (123-141) | <0.001 |
| DBP (mmHg) | | 74 (67-81) | 79 (72-86) | 85 (78-90) | 84 (78-91) | <0.001 |
| Pulse pressure (mmHg) | | 44 (38-50) | 45 (40-51) | 49 (42-55) | 48 (41-54) | <0.001 |
| Hypertension |  | 30824 (14.2) | 19185 (26.1) | 1318 (62.7) | 22816 (55.0) | <0.001 |
| Triglycerides (mmol/l) | | 0.9 (0.7-1.3) | 1.7 (1.3-2.4) | 0.9 (0.7-1.1) | 2.0 (1.4-2.7) | <0.001 |
| HDL-C (mmol/l) | | 1.5 (1.3-1.8) | 1.3 (1.1-1.5) | 1.4 (1.2-1.7) | 1.2 (1.0-1.4) | <0.001 |
| LDL-C (mmol/l) | | 3.1 (2.6-3.6) | 3.4 (2.9-4.0) | 3.1 (2.6-3.6) | 3.4 (2.9-4.0) | <0.001 |
| Use of statins |  | 6796 (3.1) | 4517 (6.1) | 281 (13.4) | 5253 (12.7) | <0.001 |
| AST (IU/L) | | 20 (17-23) | 24 (20-30) | 20 (17-23) | 26 (21-34) | <0.001 |
| ALT (IU/L) | | 19 (15-25) | 30 (22-43) | 21 (16-27) | 35 (25-53) | <0.001 |
| γ-GPT (IU/L) | | 26 (19-37) | 55 (37-89) | 24 (19-31) | 53 (36-83) | <0.001 |
| Fatty liver index* | | 14.36 (7.52-23.99) | 53.16 (44.18-65.23) | 30.80 (26.14-34.30) | 75.35 (61.87-86.71) | <0.001 |
| Visceral adiposity index** | | 0.75 (0.50-1.12) | 1.62 (1.13-2.44) | 0.82 (0.59-1.14) | 2.14 (1.45-3.15) | <0.001 |

**Supplemental table 4.**  Baseline characteristics of men classified by the presence or absence of MAFLD or MetS.

Data are presented as median (interquartile range), n (%). Analyses were performed by the Kruskal-Wallis test or Pearson’s chi-square test across groups.

MAFLD, metabolic dysfunction-associated fatty liver disease; MetS, metabolic syndrome; BMI, mass index; SBP, systolic blood pressure; DBP,: diastolic blood pressure; HDL-C, high-density lipoprotein cholesterol; LDL-C, low-density lipoprotein cholesterol; gamma-glutamyl transferase, γ-GTP; WC, waist circumference.

*: Fatty liver index: (e ^0.953*loge (TG) + 0.139*BMI + 0.718*loge (γ-GTP) + 0.053*WC - 15.745^) / (1 + e ^0.953*loge (TG) + 0.139*BMI + 0.718*loge (γ-GTP) + 0.053*WC - 15.745^) * 100

**: Visceral adiposity index: Women; [WC/ (36.58 + (1.89 × BMI))] × [TG/0.81] × [1.52/HDL-C], Men; [WC/ (39.68 + (1.88 × BMI))] × [TG/1.03] × [1.31/HDL-C] WC: waist circumference. TG: triglycerides.
